# *Dactylogyrus barnae* sp. n. (Platyhelminthes: Monogenoidea) infecting gills of *Barilius barna* Hamilton, 1822 (Pisces: Cyprinidae) from a global biodiversity hotspot - Arunachal Pradesh (India)

**DOI:** 10.14202/vetworld.2017.505-509

**Published:** 2017-05-10

**Authors:** Leki Wangchu, Dobiam Narba, Michi Yassa, Amit Tripathi

**Affiliations:** Department of Zoology, Rajiv Gandhi University, Itanagar - 791 112, Arunachal Pradesh, India

**Keywords:** Arunachal Pradesh, *Barilius barna*, *Dactylogyrus barnae* sp. n, monogenoidea, sclerotized plate

## Abstract

**Aim:**

This study was a part of an ongoing parasitological survey to investigate the health status of hill stream fish in river systems of Arunachal Pradesh - A global biodiversity hotspot.

**Materials and Methods:**

During the 2013/2015, 18 live specimens of *Barilius barna* (Cyprinidae) were captured from the local rivers of Arunachal Pradesh and examined for parasitic monogenoids. These fish, with their flatworms, were immediately fixed in hot (60°C) 4% formalin for later examination. Identification and morphometric description used in this study followed Gussev (1976). Type specimens were deposited in the British Natural History Museum, UK.

**Results:**

*Dactylogyrus barnae* sp. n. is described and illustrated from specimens of *B. barna* (Hamilton, 1822) from Arunachal Pradesh, India. The new species is characterized by a combination of the following characters: Copulatory tube coiled in 1½ counterclockwise rings, vagina consisting of a vaginal tube and vaginal pore, a complex sclerotized plate of unknown function in between male copulatory organ and vagina, and an anteromedial knob-like process on the dorsal bar.

**Conclusion:**

*D. barnae* sp. n. is the fourth species of *Dactylogyrus* described from the Northeast India and brings the total number of species of *Dactylogyrus* in Indian waters to 56. *B. barna* represents a new host record for *Dactylogyrus* spp., and possibly the first report for any parasite.

## Introduction

Monogenoidea (adj. monogenoidean) is a class of phylum Platyhelminthes, the member species of which are primarily ectoparasitic usually infecting the gills and/or external surfaces of freshwater and marine fishes. *Dactylogyrus* Diesing, 1850 is one the largest genera of the monogenoidea with more than 900 species described to date [1] of which 55 species have been described from India [2]. More than 95% species of *Dactylogyrus* are parasites of the cyprinid fish [1] and are known to be significant pathogens, producing chronic debility, poor development and growth, impaired respiration, and finally mass mortality of infested hosts [3,4].

Arunachal Pradesh (29°30’N; 97°30’E) is recognized as the 25^th^ biodiversity hotspot in the world [5] and among the 200 globally important ecoregions [6]. The region is also identified as one of the global hotspots of fishery resources [7] with 213 plus recorded fish species [8] representing 23.43% of the total Indian freshwater fish species. Not only does these fish provide nutritious food, but also forms an unbreakable relationship with the culture, religion, and traditions of the region [9]. However, unfortunately, the information on fish parasites of this region is poorly documented [10]. As such, the intensification and development of freshwater aquaculture in Arunachal Pradesh urgently require research knowledge and expertise on fish diseases and fish health protection.

*Barilius barna* (Hamilton, 1822) is one of the commonly exported indigenous species of ornamental fish of the Northeast India [11] and has a commercial importance too [12]. During an ongoing parasitological survey of hill stream fish of Arunachal Pradesh, *B*. *barna*, which has not previously been examined for parasitic monogenoids, was found infected with a new species of *Dactylogyrus*, which is described and illustrated in this paper.

## Materials and Methods

### Ethical approval

This work received ethical approval from the Institutional Ethics Committee of Rajiv Gandhi University, Itanagar, Arunachal Pradesh.

### Experimental design

**Figure-1:**
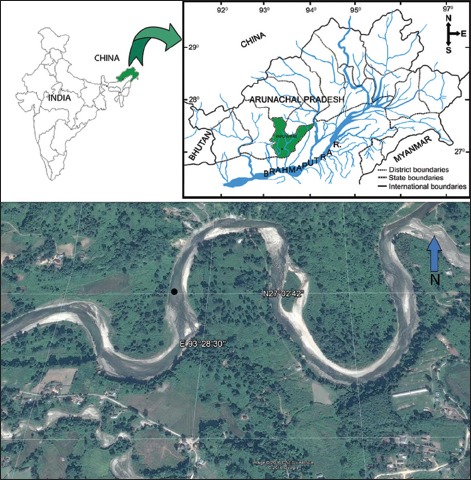
Collection site (type locality) of *Barilius barna* in Arunachal Pradesh, India (satellite image from Google Earth™).

During February 2013 to March 2015, 18 live specimens of *B. barna* were collected from three Rivers - the Jote, Tezu, and Dihang - in Arunachal Pradesh, India with the help of gill nets (Figure-1). Their gills were surgically removed and immediately fixed in 60°C 4% formalin for later examination under stereomicroscope (Leica EZ 4HD). Some of the fish samples were kept alive in plastic coolers containing river water and ice blocks before being transported to the laboratory where they were maintained in glass aquaria supplied with aerator. Monogenoids were picked off the gills using fine needles, stained and mounted according to the procedures recommended by Kritsky *et al*. [13]. The mounted parasites were photographed with a digital camera (ProgRes CapturePro v2.8.8) attached to a microscope equipped with phase-contrast optics (Olympus CX41). Based on these photographs, illustrations were drawn on a digitizing tablet (WACOM) using Adobe Illustrator software, and the measurements (in micrometers) were obtained with the software ProExpress 6.0 (Media Cybernetics, Inc., USA); the mean is followed by the range and number of specimens measured (n) in parentheses. Identification and morphometric characteristics followed Gussev [14]. The direction of the coil (clockwise vs. counterclockwise) of the copulatory tube was determined using the procedure suggested by Kritsky *et al*. [15]. Fish name followed that provided in FishBase [12]. Holotype and paratypes were deposited in the British Natural History Museum, UK. The prevalence (percentage of infected hosts in a sample) and mean intensity (mean number of parasites per infected host in a sample) of infection were calculated according to Bush *et al*. [16].

## Results and Discussion

- Phylum: Platyhelminthes
- Class: Monogenoidea Bychowsky, 1937
- Order: Dactylogyridea Bychowsky, 1937
- Family: Dactylogyridae Bychowsky, 1933
- Type host: *B. barna* (Hamilton, 1822) (Cypriniformes, Cyprinidae)
- Infection site: Gills
- Type locality: River Jote, Papum Pare district, Arunachal Pradesh, India (27°2’N/93°28’E)
- Additional locality: River Tezu, Lohit district (27°55’N/96°10’E), and River Dihang, Roing district (28°8’N/95°50’E) at Arunachal Pradesh, India
- Prevalence: 100% (examined 18, infected 18)
- Mean intensity: 35 parasites per infected host
- Type material: Holotype (NHMUK 2013.8.28.1); nine paratypes (NHMUK 2013.8.28.2-10) in British Natural History Museum, UK
- Etymology: The specific epithet refers to the species of the type host.

### Description (Figures-2a-h and 3)

**Figure-2:**
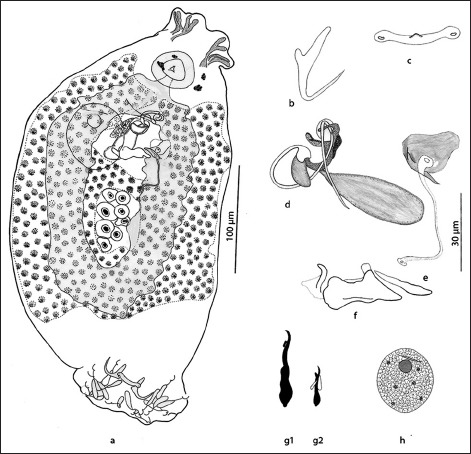
*Dactylogyrus barnae* sp. n. from *Barilius barna* (Hamilton, 1822). (a) Whole mount, (composite drawing, dorsal view), (b) dorsal anchor, (c) dorsal bar, (d) male copulatory organ (ventral view) with prostatic reservoirs, (e) vagina, (f) sclerotized plate, (g1) hook pairs 1-5 and 7, (g2) hook pair 6 with 4A, (h) egg (figure a and h scale to individual 100 µm bars. Remaining figures scale to 30 µm bar).

**Figure-3:**
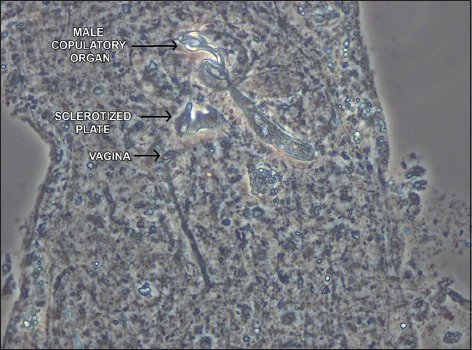
Male copulatory organ, complex sclerotized plate, and vagina of *Dactylogyrus barnae* sp. n.

Body 478 (443-513; n=9) long, fusiform; greatest width 191 (157-226; n=9) in trunk region. Cephalic lobes well developed, two pairs; head organs conspicuous, four pairs. Two pairs of eyespots, posterior pair larger, anterior pair closer together, accessory pigment granules present or absent in cephalic and anterior trunk regions. Pharynx ovate, 33 (23-41; n=7) long, 25 (22-32; n=7) wide.

Testis and vas deferens not observed; seminal vesicle a simple dilation of vas deferens. Male copulatory organ sclerotized and composed of copulatory tube and accessory piece. Copulatory tube 26 (22-30; n=7) long, comprising coil of approximately 1½, counterclockwise rings, inflated base. Accessory piece 19 (14-24; n=7) long, proximal part articulates with base of copulatory tube, distal part serves to guide the copulatory tube. Pair of prostatic reservoirs opens into base of copulatory tube. A complex sclerotized plate comprising a flat median and two rod-like lateral structures in between male copulatory organ and vagina. Ovary oval, 60 (48-68; n=28) long, 30 (26-37; n=28) wide, intercecal; oviduct, ootype, and uterus not observed. Vagina consists of vaginal pore and vaginal tube, lightly sclerotized, dextral to midline, immediately above the seminal receptacle. Vitelline follicles dense, present throughout trunk, except in regions of reproductive organs. Egg 33 (32-40; n=5) long, 28 (25-33; n=5) wide, oval, apparently without polar filament.

Haptor 59 (50-67; n=9) long, 85 (73-96; n=9) wide. Dorsal anchors 29 (27-30; n=7) long, with well-developed inner roots 13 (12-15; n=7) long, recurved point straight, extending past level of shank, 16 (15-18; n=7) long, outer roots 4 (3-5; n=7) long. Dorsal bar 27 (23-31; n=8) long, slightly curved backward, with dilated, fenestrated ends, and an anteromedial knob-like process. 7 pairs of hooks similar in shape but dissimilar in size: Pairs 1-5 and 7, 24 (21-28; n=9) long, with depressed thumb; pair 6, 13 (12-14; n=7) long, with protruding thumb and associated 4A.

### Remarks

*Dactylogyrus barnae* sp. n. is characterized by a combination of the following characters: Copulatory tube coiled in 1½ counterclockwise rings, vagina consisting of vaginal tube and vaginal pore, a complex sclerotized plate of unknown function in between male copulatory organ and vagina, and an anteromedial knob-like process on the dorsal bar. The presence of a complex sclerotized plate is the most distinguishing feature of the new species. Kritsky *et al*. [13] showed a similar structure in the member species of *Urocleidoides* Mizelle and Price, 1964 to which they named as vaginal sclerite since it overhung vagina. In this case, however, the sclerotized plate was almost always found in between male copulatory organ and vagina, which makes it difficult for us to predict its function. *D*. *barnae* differs from its closest apparent relative, *D*. *parvianchoris* Gusev, 1976 from *Salmostoma bacaila* (Hamilton, 1822) (Cypriniformes: Cyprinidae), most notably by having small outer roots in dorsal anchors (absent in *D*. *parvianchoris*), fenestrated ends, and an anteromedial knob-like process in dorsal bar (absent in *D*. *parvianchoris*), and a complex sclerotized plate in between male copulatory organ and vagina (absent in *D*. *parvianchoris*).

*D*. *barnae* sp. n. is the fourth species of *Dactylogyrus* described from the Northeast India, and brings the total number of species of *Dactylogyrus* in Indian waters to 56. Incidentally, the new species is only the sixth record of parasitic monogenoids from Arunachal Pradesh. Earlier, Porwal *et al*. [17] described two new species of *Ancyrocephalus* Creplin, 1839 (*Acanthocobitis botia* and *Abietinaria cruciformis*) from the gills of mottled loach *A. botia* (Hamilton, 1822) (Cypriniformes: Nemacheilidae); Narba and Wangchu [18] described three new species of *Dactylogyrus* Diesing, 1850 (*Dactylogyrus nasutai*, *Dactylogyrus yachuliensis*, and *Dactylogyrus siangensis*) from the gills of *Garra nasuta* (McClelland, 1838) and *Bangana dero* (Hamilton, 1822) (Cypriniformes: Cyprinidae); Wangchu and Narba [19] described two new species of *Thaparocleidus* Jain, 1952 (*Thaparocleidus motumensis* and *Thaparocleidus pterocryptisii*) from *Pterocryptis indicus* (Datta, Barman and Jayaram, 1987) (Siluriformes: Siluridae); Yassa and Wangchu [20] provided preliminary observations on a possibly new genus from the gills of Gangetic loach *Botia rostrata* (Gunther, 1868) (Cypriniformes: Cobitidae); Tripathi *et al*. [21] recorded *Dactylogyrus sphyrnoides* Gussev, 1976, on a near threatened Tor Barb, *Tor tor* (Hamilton, 1922).

This paucity of studies on parasitic monogenoids in Arunachal Pradesh means there is a lack of baseline data about levels of parasitism on fish populations and it could possibly be assigned to a host of reasons, notably, the remoteness of the region, difficult terrain and, most importantly, “taxonomic impediment” - Lack of parasite taxonomists - in the region. The state of Arunachal Pradesh is predominantly characterized by topographical constraints such as altitudinal and geographical variations, mountain slopes, and infertile soil, which are not suited for the high level of agricultural production [22]. This imbalance can however be removed by the adequate parasitological research and development support in the fishery sector since the improvement of yield can mainly be achieved from a healthy stock. It is worth mentioning here that monogenoids are very difficult to be eradicated once they are introduced in the aquatic system. A number of simple chemicals (e.g., hydrogen peroxide, potassium permanganate, and ammonium chloride, and sodium chloride), staining dyes (e.g., malachite green, methylene blue, acriflavine), antimalarial (quinine hydrochloride and atebrine hydrochloride), drugs (e.g., praziquantel and mebendazole), and even formaldehyde have been effectively used to control the monogenoids with varying success [23,24], but they can be very expensive [25]. Clearly, there is a pressing need for bringing the parasitic monogenoids of Arunachal Pradesh to the attention of the fish biologists, ichthyologists and parasitologists.

*B. barna* represents a new host record for *Dactylogyrus* spp. In fact, a transparent and reproducible literature exploration, including peer reviewed journals, bibliographic databases and web searches, suggests that this is also the first report of any parasite from *B*. *barna* since there are not any studies investigating the parasites in this fish.

## Conclusion

*Dactylogyrus barnae* sp. n. is described and illustrated from specimens of *Barilius barna* (Hamilton, 1822) (Cyprinidae) collected from Arunachal Pradesh (India) - a global biodiversity hotspot. The new species is characterized by a combination of the following characters: Copulatory tube coiled in 1½ counterclockwise rings, vagina consisting of vaginal tube and vaginal pore, a complex sclerotised plate of unknown function in between male copulatory organ and vagina, and an anteromedial knob-like process on dorsal bar. *D*. *barnae* sp. n. is the fourth species of *Dactylogyrus* described from the northeast India, and brings the total number of species of *Dactylogyrus* in Indian waters to 56. *Barilius barna* represents a new host record for *Dactylogyrus* spp., and possibly the first report for any parasite. Arunachal Pradesh has rich freshwater fishery resources, and yet the region has a distinct lack of baseline parasite data. As such, there is a distinct need for further detailed studies of fish parasites in Arunachal Pradesh to provide better management and conservation of fishery resources.

## Authors’ Contributions

LW and AT conceived and designed the experiment work. LW and DN collected the host specimens. DN and MY performed the experiments and prepared the drawings of parasite specimen. AT drafted and revised the manuscript. All authors read and approved the final manuscript.

## References

[1] Gibson D.I, Timofeeva T.A, Gerasev P.I (1996). A catalogue of the nominal species of the monogenean genus *Dactylogyrus* diesing, 1850 and their host genera. Syst Parasitol.

[2] Pandey K.C, Agrawal N (2008). An encyclopedia of Indian Monogenoidea.

[3] Bauer O.N (1951). Concerning pathogenicity of *Dactylogyrus solidus* achmerov. Doklady Akad Nauk. USSR.

[4] Paperna I (1963). Dynamics of *Dactylogyrus vastator* population on carp fry gills. Bamidgeh Bull. Fish Cult. Isr.

[5] Chowdhery H.J, Mudgal V, Hajra PK (1999). Arunachal Pradesh;in floristic diversity and conservation strategies in India. The Context of States and Union Territories.

[6] Olson D.M, Dinerstein E (1998). The global 200: A representation approach to conserving the earth's most biologically valuable ecoregions. Conserv. Biol.

[7] Kottelat M, Whitten T (1996). Freshwater biodiversity in Asia with special reference to fish. World Bank Tech. Paper No. 343.

[8] Bagra K, Kadu K, Sharma K.N, Laskar B.A, Sarkar U.A, Das D.N (2009). Ichthyological survey and review of the checklist of fish fauna of Arunachal Pradesh, India. Check List.

[9] Gurumayum S.D, Devi G.A, Nandeesha M.C, Poo P.S, Hall S.J, Williams M.J (2006). Women's participation in fisheries activities in Manipur Valley in India with traditional fish-based beliefs and customs. Global Symposium on Gender and Fisheries.

[10] Tripathi A (2011). Helminth richness in Arunachal Pradesh fishes: A forgotten component of biodiversity. J. Biosci.

[11] Jayalal L, Ramachandran A (2012). Export trend of Indian ornamental fish industry. Agric. Biol. J. N. Am.

[12] Froese R, Pauly D (2014). Fish Base. World Web Electronic Publication.

[13] Kritsky D.C, Thatcher V.E, Boeger W.A (1986). Neotropical monogenea i8. revision of urocleidoides (Dactylogyridae, Ancyrocephalinae). Proc. Helminthol. Soc. Wash.

[14] Gussev A.V (1976). Freshwater Indian monogenoidea. Principles of systematics, analysis of the world faunas and their evolution. Indian J. Helminthol.

[15] Kritsky D.C, Boeger W.A, Thatcher V.E (1985). Neotropical monogenea 7. Parasites of the pirarucu *Arapaima gigas* (Cuvier), with descriptions of two new species and redescripton of *Dawestrema cycloancistrium* price and nowlin, 1967 (Dactylogyridae: Ancyrocephalinae). Proc. Biol. Soc. Wash.

[16] Bush A.O, Lafferty K.D, Lotz J.M, Shostak A.W (1997). Parasitology meets ecology on its own terms: Margolis et al revisited. J. Parasitol.

[17] Porwal S, Agrawal N, Pandey K.C, Tripathi A, Tripathi A (2012). Two new species of *Ancyrocephalus* (S.L.)Gussev, 1976 (Monogenoidea: Dactylogyridae) from the gills of mottled loach *Acanthocobitis botia* (Cypriniformes *Balitoridae*) from India. Proceeding of the 1^st^ National Symposium on Fish Parasites, 72-77. Itanagar, March 19-20-2012.

[18] Narba D, Wangchu L, Tripathi A (2015). Three new species of *Dactylogyrus* diesing, 1850 (Monogenoidea: Dactylogyridae) from gills of *Garra nasuta* and *Bangana dero* (Cypriniformes *Cyprinidae*) from Arunachal Pradesh. of the 2^nd^ National Symposium on Fish Parasites 43-49 Itanagar November 23-24-2015.

[19] Wangchu L, Narba D, Tripathi A (2015). A preliminary survey of parasitic monogenoids (Platyhelminthes) of some important catfish (Teleostei: Siluriformes) in Arunachal Pradesh, with description of two new species of *Thaparocleidus* Jain 1952. Proceeding of the 2^nd^ National Symposium on Fish Parasites, 43-49. Itanagar, November 23-24, 2015.

[20] Yassa M, Wangchu L, Tripathi A (2015). Preliminary observations on a new genus of parasitic monogenoid from *Botia rostrata* (Gunther 1868) in Arunachal Pradesh. Proceeding of the 2 nd National Symposium on Fish Parasites 43-49 Itanagar November 23-24 2015.

[21] Tripathi A, Wangchu L, Narba D (2016). New host and distribution records of the gill parasite *Dactylogyrus sphyrnoides* Gussev, 1976 (Platyhelminthes, Monogenoidea) on a near threatened tor barb *Tor tor* (Hamilton 1822) (Teleostei *Cyprinidae*), in northeastern India. Check List.

[22] New Agricultural Policy (2015). Official Website of Government of Arunachal Pradesh.

[23] Schmahl G (1991). The chemotherapy of monogeneans, which parasitize fish: A review. Folia Parasitol.

[24] Reed P, Francis-Floyd R, Klinger R, Petty D (2009). Monogenean parasites of fish. Fisheries and Aquatic Sciences.

[25] Whittington I.D, Ernst I, Corneillie S, Talbot C (2001). Sushi, fish, and parasites. Australas. Sci.

